# Development and validation of a prognostic model for patients with hepatocellular carcinoma undergoing radiofrequency ablation

**DOI:** 10.1002/cam4.2417

**Published:** 2019-07-10

**Authors:** Chang Gon Kim, Hyun Woong Lee, Hye Jin Choi, Jung Il Lee, Hye Won Lee, Seung Up Kim, Jun Yong Park, Do Young Kim, Sang Hoon Ahn, Kwang‐Hyub Han, Han Sang Kim, Kyung Hwan Kim, Seong Jin Choi, Yongun Kim, Kwan Sik Lee, Gyoung Min Kim, Man Deuk Kim, Jong Yoon Won, Do Yun Lee, Beom Kyung Kim

**Affiliations:** ^1^ Department of Internal Medicine Yonsei University College of Medicine Seoul Republic of Korea; ^2^ Graduate School of Medical Science and Engineering Korea Advanced Institute of Science and Technology Daejeon Republic of Korea; ^3^ Institute of Gastroenterology Yonsei University College of Medicine Seoul Republic of Korea; ^4^ Yonsei Liver Center Severance Hospital Seoul Republic of Korea; ^5^ Department of Radiology Yonsei University College of Medicine Seoul Republic of Korea

**Keywords:** carcinoma, hepatocellular, disease‐free survival, nomograms, prognosis, radiofrequency ablation

## Abstract

**Background:**

There are large variations in prognosis among hepatocellular carcinoma (HCC) patients undergoing radiofrequency ablation (RFA). However, current staging or scoring systems hardly discriminate the outcome of HCC patients treated with RFA.

**Methods:**

A total of 757 treatment‐naïve HCC patients undergoing RFA (derivation cohort) were analyzed to establish a nomogram for disease‐free survival (DFS) based on Cox proportional hazard regression model. Accuracy of the nomogram was assessed and compared with conventional staging or scoring systems. Furthermore, external validation was performed in an independent cohort including 208 patients (validation cohort).

**Results:**

Tumor size, tumor number, alpha‐fetoprotein, prothrombin induced by vitamin K absence‐II, lymphocyte count, albumin, and presence of ascites were adopted to construct the prognostic nomogram from the derivation cohort. Calibration curves to predict probability of DFS at 3 and 5 years after RFA showed good agreements between the nomogram and actual observations. The concordance index of the present nomogram was 0.759 (95% confidence interval 0.728‐0.790), which was superior to those of conventional staging or scoring systems (range 0.505‐0.683, all *P* < .001). These results were also reproduced in the validation cohort.

**Conclusion:**

Our simple‐to‐use nomogram optimized for treatment‐naïve HCC patients undergoing RFA provided better prognostic performance than conventional staging or scoring systems.

## 1 INTRODUCTION

Hepatocellular carcinoma (HCC) has been ranked as the sixth most common malignancy and the fourth most frequent cause of cancer‐related death in the world.^1^ Hepatic resection or liver transplantation is the preferred curative treatment modality for patients with HCC.^2^ However, only a minor portion could become eligible candidates at the time of diagnosis due to various reasons such as limited hepatic functional reserve, shortage of organ donor, high morbidities and mortalities accompanied by surgery, and patients' refusal.^3^ Thus, various approaches have been developed as nonsurgical treatment options.^4^, ^5^ Among these, percutaneous radiofrequency ablation (RFA) is recommend as a first‐line treatment in very early stage HCC (single tumor with a diameter <2 cm) and an alternative first‐line treatment in early stage HCC (up to three HCC with a maximal diameter <3 cm), specifically in light of its lesser invasiveness and better tolerability compared to hepatic resection.^6^ Furthermore, over the several decades, primarily owing to the advances in RFA techniques, more reliable tumor control with lesser complications has been achieved.^7^ However, the high rates of postprocedural recurrence (either local or distant), which might be up to 70% at 5 years, remain a major challenge for long‐term survival.^8^

Several staging or scoring systems had been proposed to predict survival and to guide treatment strategies in patients with HCC, so far. Nevertheless, their performances to predict the prognosis among those treated with RFA have been largely limited, because such systems were originally designed to cover the full spectrum of HCC patients (from very early to far advanced stage cases), rather than “very early or early stage” HCC patients.^9^ In addition, individual‐based comprehensive analysis using robust clinical parameters including classical tumor factors (eg, size or number), biomarkers, hepatic reserve, and other host factors is still scarce.

In this study, we aimed to establish a prognostic nomogram to predict postprocedural outcomes in HCC patients treated with RFA as a first‐line treatment and to externally validate its predictive performance in an independent cohort.

## 2 MATERIALS AND METHODS

### 2.1 Derivation and validation cohort

Between February 2005 and December 2014, treatment‐naïve patients with HCC who underwent RFA as an initial treatment at Severance Hospital, Seoul, Republic of Korea, were considered eligible for the derivation cohort. Inclusion criteria were as follows: age ≥ 19 years old; at least one unidimensional lesion measurable according to modified Response Evaluation Criteria in Solid Tumors (mRECIST)^10^; tumor size up to 5 cm; tumor number up to 3; Eastern Cooperative Oncology Group performance status of 0‐2; platelet count ≥ 50 × 10^3^/µL; serum aspartate aminotransferase (AST) or alanine aminotransferase (ALT) level < 10 times the upper limit of normal. Exclusion criteria were as follows: the presence of portal or hepatic vein invasion; the presence of extrahepatic spread; Child‐Pugh class C; any other uncontrolled comorbidities or malignant neoplasm; and a prior liver transplant. During the same period, treatment‐naïve patients with HCC who underwent RFA as an initial treatment at Gangnam Severance Hospital, Seoul, Republic of Korea, were considered eligible for the independent validation cohort. The same inclusion and exclusion criteria were applied.

The study was performed in accordance with the Declaration of Helsinki and Good Clinical Practice and was approved by the institutional review boards (4‐2018‐0969). Informed consent for the invasive procedures was acquired from all patients.

### 2.2 Data collection

The collected clinical data were as follows: patients' baseline clinical features before the primary RFA (age, gender, laboratory findings including alpha‐fetoprotein [AFP] and prothrombin induced by vitamin K absence‐II [PIVKA‐II], tumor etiologies, and hepatic functional reserve) and primary tumor factors such as tumor number and tumor size. Neutrophil‐to‐lymphocyte ratio (NLR) was calculated by dividing absolute neutrophil count by lymphocyte count. Albumin‐bilirubin (ALBI) grade was calculated based on a previous report.^11^ Furthermore, American Joint Committee on Cancer stage,^12^ Barcelona Clinic Liver Center stage,^13^ Cancer of the Liver Italian Program score,^14^ Chinese University Prognostic Index,^15^ Okuda stage,^16^ Japan Integrated Staging (JIS) score,^17^ and Group d'Etude ed te Traitement du Carcinome Hepatocellular^18^ were assessed in each patient (Table S1).

### 2.3 RFA procedure and follow‐up

The diagnosis of HCC was confirmed based on current practice guidelines of the American Association for the Study of Liver Diseases and the European Association for the Study of the Liver.^4^, ^19^ Planning ultrasound was routinely performed to assess the feasibility and applicability for RFA. Experienced interventional radiologists performed the RFA procedures. Briefly, patients were treated percutaneously using an RFA device under ultrasound guidance. The tumor was ablated until complete ablation of the entire tumor was achieved. Immediately after RFA, complete ablation was confirmed with a dynamic CT scan. Laboratory tests including tumor markers (AFP, PIVKA‐II) and imaging studies (dynamic CT or MRI) were performed 1 month post‐RFA and every 3 months thereafter to monitor tumor recurrence.

### 2.4 Statistical analysis

Variables are expressed as the median (interquartile range [IQR]), or number (%) as appropriate. Differences among continuous and categorical variables were examined for statistical significance with Student's *t *test (or Mann‐Whitney test, if appropriate) and chi‐squared test (or Fisher's exact test, if appropriate). The primary endpoint used for a model development was disease‐free survival (DFS), defined as the time between RFA and recurrence, death from any cause, or the last date of follow‐up. DFS was calculated using Kaplan‐Meier analysis and compared by log‐rank test. To identify the factors associated with DFS in the derivation cohort, Cox‐regression model was used to calculate the hazard ratio (HR) and 95% confidence interval (CI) of each variable on univariate and multivariate analyses. Factors significantly associated with DFS on multivariate analysis were adopted to construct a nomogram and the weighted risk score of each variable in the model was calculated based on *β*‐regression coefficient by Cox‐regression model.

The performance of a novel prognostic model to predict the probability of DFS at 6‐60 months after RFA was estimated as integrated area under the curve (iAUC). For calibration, bootstraps with 1000 resamples were used to compare actual survival and predicted survival derived from the prognostic model at 3 and 5 years after RFA. Furthermore, the overall prognostic performance of the prognostic model was assessed using the concordance index (c‐index). The c‐index was compared between our nomogram and other conventional staging or scoring systems. For external validation, similar methods were applied for the independent cohort.

*P* < .05 was considered statistically significant. All statistical analyses were performed using R software (version 3.5.0., http://cran.r-project.org/).

## 3 RESULTS

### 3.1 Patients' baseline characteristics and overall prognosis in the derivation cohort

In total, 757 patients were included in the derivation cohort. Baseline clinical characteristics are described in the Table 1. The median follow‐up duration was 39.8 months, the median age was 63 years, and 77.3% were male. Chronic hepatitis B virus (HBV) infection was the most common etiology, accounting for more than 75%. The median tumor size was 1.8 cm (IQR 1.4‐2.4 cm). The median values of AFP and PIVKA‐II were 7.9 ng/mL (IQR 3.2‐44.1 ng/mL) and 29 mAU/mL (IQR 19‐55 mAU/mL), respectively. During follow‐up, 400 patients (52.8%) experienced HCC recurrence after RFA in the derivation cohort. The DFS rates at 3 and 5 years were 41.4% and 31.5%, respectively.

**Table 1:** Patient characteristics of the derivation and validation cohorts

Variables	Derivation cohort (n = 757)	Validation cohort (n = 208)	*P* value
Age			.102
Median (IQR), years	63 (55‐69)	60 (54‐68)	
Sex			.206
Male	585 (77.3%)	152 (73.1%)	
Female	172 (22.7%)	56 (26.9%)	
Etiology			.928
HBV	560 (74.0%)	150 (72.1%)	
HCV	123 (16.2%)	36 (17.3%)	
HBV, HCV	9 (1.2%)	2 (1.0%)	
NBNC	65 (8.6%)	20 (9.6%)	
Tumor size			.749
<2 cm	407 (53.8%)	116 (55.8%)	
2‐3 cm	260 (34.3%)	71 (34.1%)	
≥3 cm	90 (11.9%)	21 (10.1%)	
Tumor numbers			.945
1	644 (85.1%)	175 (84.1%)	
2	82 (10.8%)	24 (11.5%)	
3	31 (4.1%)	9 (4.3%)	
AFP			.218
<20 ng/mL	490 (64.7%)	125 (60.1%)	
≥20 ng/mL	267 (35.3%)	83 (39.9%)	
PIVKA‐II			.381
<40 mAU/mL	505 (66.7%)	132 (63.5%)	
≥40 mAU/mL	252 (33.3%)	76 (36.5%)	
Neutrophil			.377
<4000/µL	227 (30.0%)	69 (33.2%)	
≥4000/µL	139 (70.0%)	139 (66.8%)	
Lymphocyte			.163
<2000/µL	484 (63.9%)	122 (58.7%)	
≥2000/µL	273 (36.1%)	86 (41.3%)	
Anemia			.097
Presence	217 (28.7%)	72 (34.6%)	
Absence	540 (71.3%)	136 (65.4%)	
Platelet			.693
<150 × 10^3^/µL	567 (74.9%)	153 (73.6%)	
≥150 × 10^3^/µL	190 (25.1%)	55 (26.4%)	
Bilirubin			.814
<2 mg/dL	720 (95.1%)	197 (94.7%)	
≥2 mg/dL	37 (4.9%)	11 (5.3%)	
Albumin			.839
≤3.5 g/dL	231 (30.5%)	65 (31.2%)	
>3.5 g/dL	526 (69.5%)	143 (68.8%)	
Ascites			.959
Presence	81 (10.7%)	22 (10.6%)	
Absence	676 (89.3%)	186 (89.4%)	
AST			.424
<40 IU/L	373 (49.3%)	109 (52.4%)	
≥40 IU/L	384 (50.7%)	99 (47.6%)	
ALT			.780
<40 IU/L	625 (82.6%)	170 (81.7%)	
≥40 IU/L	132 (17.4%)	38 (18.3%)	
ALP			.841
<143 IU/L	703 (92.9%)	194 (93.3%)	
≥143 IU/L	54 (7.1%)	14 (6.7%)	

Abbreviations: AFP, alpha‐fetoprotein; ALP, alkaline phosphatase; ALT, alanine aminotransferase; AST, aspartate aminotransferase; HBV, hepatitis B virus; HCV, hepatitis C virus; IQR, interquartile range; NBNC, non‐B, non‐C; PIVKA‐II, prothrombin induced by vitamin K absence‐II.

### 3.2 Development of the prognostic nomogram and its performance

**Figure 1:**
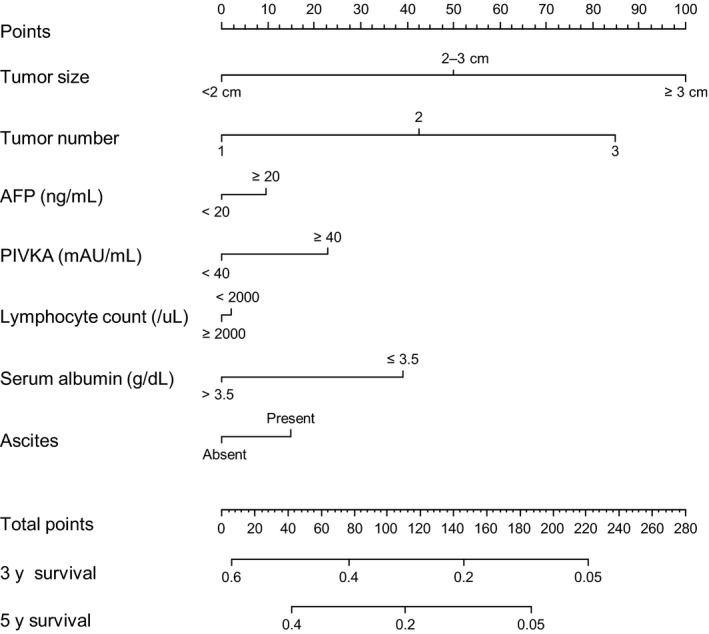
Nomogram for disease‐free survival in patients with hepatocellular carcinoma treated with radiofrequency ablation. AFP, alpha‐fetoprotein; PIVKA, prothrombin induced by vitamin K absence‐II.

Fifteen variables were assessed on univariate analysis to identify significant factors associated with DFS in the derivation cohort. Subsequently, variables with *P* < .05 on univariate analysis including etiology, tumor size, tumor number, AFP, PIVKA‐II, neutrophil count, lymphocyte count, anemia, platelet count, bilirubin, albumin, ascites, AST, ALT, and ALP (alkaline phosphatase) levels were entered into a multivariate Cox‐regression model. Finally, seven essential variables including tumor size, tumor number, AFP level, PIVKA‐II level, lymphocyte count, albumin level, and presence of ascites were identified as prognostic factors with statistical significance (Table 2). Based on *β*‐regression coefficients calculated from the final Cox‐regression model, a weighted risk score with a rounded form to the integer value was allocated to each variable to construct a prognostic nomogram (Table 3). The prognostic nomogram was expressed as the total sum of each risk score (Figure 1).

**Table 2:** Factors associated with disease‐free survival in the derivation cohort

Variables	Univariate analysis		Multivariate analysis	
	HR (95% CI)	*P* value	Adjusted HR (95% CI)	*P* value
Age		.229		
<65 years	Ref			
≥65 years	1.129 (0.927‐1.375)			
Sex		.239		
Male	1.151 (0.911‐1.454)			
Female	Ref			
Etiology		.008		.307
HCV	1.338 (1.090‐1.768)		1.144 (0.883‐1.482)	
Non‐HCV	Ref		Ref	
Tumor size		<.001		<.001
<2 cm	Ref		Ref	
2‐3 cm	2.061 (1.654‐2.569)		1.868 (1.488‐2.345)	
≥3 cm	6.562 (4.958‐8.686)		5.100 (3.780‐6.881)	
Tumor numbers		<.001		<.001
1	Ref		Ref	
2	2.291 (1.705‐3.077)		1.581 (1.156‐2.163)	
3	4.887 (3.337‐7.158)		4.007 (2.696‐5.957)	
AFP		<.001		.001
<20 ng/mL	Ref		Ref	
≥20 ng/mL	1.595 (1.307‐1.946)		1.443 (1.172‐1.777)	
PIVKA‐II		<.001		001
<40 mAU/mL	Ref		Ref	
≥40 mAU/mL	1.980 (1.625‐2.412)		1.451 (1.174‐1.793)	
Neutrophil		<.001		.419
<4000/µL	1.471 (1.198‐1.807)		1.099 (0.874‐1.383)	
≥4000/µL	Ref		Ref	
Lymphocyte		<.001		.026
<2000/µL	1.537 (1.242‐1.902)		1.332 (1.034‐1.714)	
≥2000/µL	Ref		Ref	
Anemia		<.001		.565
Presence	1.830 (1.493‐2.242)		1.074 (0.842‐1.369)	
Absence	Ref		Ref	
Platelet		.009		.604
<150 × 10^3^/µL	1.366 (1.080‐1.728)		0.928 (0.698‐1.232)	
≥150 × 10^3^/µL	Ref		Ref	
Bilirubin		.001		.859
<2 mg/dL	Ref		Ref	
≥2 mg/dL	1.891 (1.287‐2.778)		0.963 (0.632‐1.465)	
Albumin		<.001		.008
≤3.5 g/dL	2.413 (1.976‐2.946)		1.408 (1.092‐1.817)	
>3.5 g/dL	Ref		Ref	
Ascites		<.001		.001
Presence	1.740 (1.317‐2.300)		1.636 (1.209‐2.213)	
Absence	Ref		Ref	
AST		<.001		.411
<40 IU/L	Ref		Ref	
≥40 IU/L	1.742 (1.425‐2.130)		1.107 (0.869‐1.410)	
ALT		.013		.169
<40 IU/L	Ref		Ref	
≥40 IU/L	1.359 (1.066‐1.733)		1.213 (0.921‐1.597)	
ALP		<.001		.399
<143 IU/L	Ref		Ref	
≥143 IU/L	2.225 (1.626‐3.046)		1.162 (0.820‐1.647)	

Abbreviations: AFP, alpha‐fetoprotein; ALT, alanine aminotransferase; ALP, alkaline phosphatase; AST, aspartate aminotransferase; CI, confidence interval; HR, hazard ratio; HCV, hepatitis C virus; PIVKA‐II, prothrombin induced by vitamin K absence‐II; Ref, reference.

**Table 3:** β‐coefficient and risk score from multivariate Cox‐regression model in the derivation cohort

Variables	β‐coefficient	*P* value	Risk score
Tumor size		<.001	
<2 cm	Ref		0
2‐3 cm	0.635		50
≥3 cm	1.646		100
Tumor numbers		<.001	
1	Ref		0
2	0.517		42
3	1.395		85
AFP		<.001	
<20 ng/mL	Ref		0
≥20 ng/mL	0.402		9
PIVKA‐II		<.001	
<40 mAU/mL	Ref		0
≥40 mAU/mL	0.380		23
Lymphocyte		.010	
<2000/µL	0.287		0
≥2000/µL	Ref		2
Albumin		<.001	
≤3.5 g/dL	0.444		0
>3.5 g/dL	Ref		39
Ascites		.001	
Presence	0.469		0
Absence	Ref		15

Abbreviations: AFP, alpha‐fetoprotein; PIVKA‐II, prothrombin induced by vitamin K absence‐II; Ref, reference.

**Figure 2:**
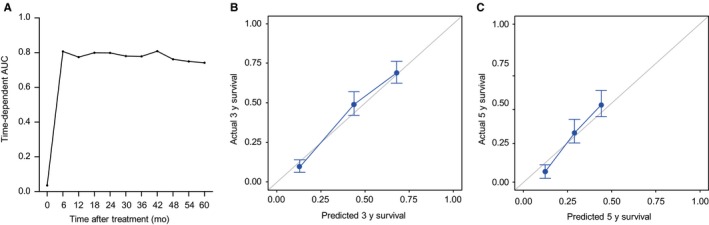
Performance power of nomogram in the derivation cohort. A, Area under the curves (AUCs) for the prediction of disease‐free survival (DFS) at 6‐60 months after radiofrequency ablation among the derivation cohort. Calibrations of the nomogram for the prediction of DFS at (B) 3 y and (C) 5 y among the derivation cohort

The proposed prognostic nomogram showed a fairly good discrimination capability to predict DFS at 6‐60 months after RFA, with iAUC of 0.780 (Figure 2A). Furthermore, the actual DFS was in good agreement with the predicted DFS at 3 years (Figure 2B) and 5 years (Figure 2C). The overall c‐index of the prognostic model was 0.759 (95% CI 0.728‐0.790) and was higher than those of the conventional staging or scoring systems (Table S2).

**Figure 3:**
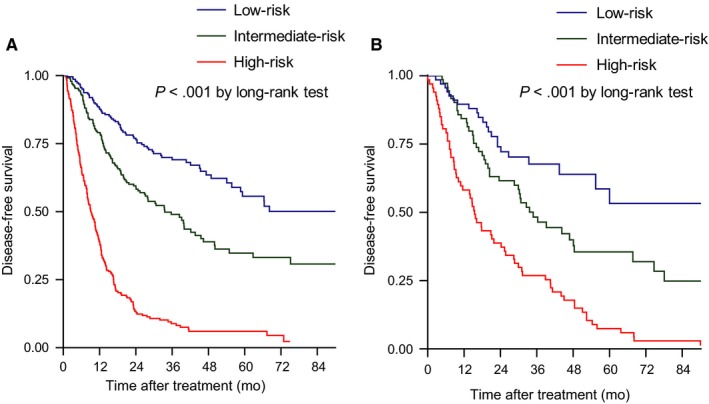
Kaplan‐Meier curves according to the risk stratification by the prognostic nomogram among the derivation (A) and validation (B) cohorts

Patients were stratified into three groups (low‐, intermediate‐, and high‐risk groups) according to the tertile of the total sum of allocated scores from the prognostic nomogram; the median DFS rates of low‐, intermediate‐, and high‐risk groups were 106.3, 33.6, and 9.3 months, respectively (*P* < .001 by log‐rank test, Figure 3A).

### 3.3 Recruitment of the independent validation cohort and external validation of the prognostic nomogram

A total of 208 patients were included in the validation cohort. As shown in Table 1 and Table S1, patients' baseline characteristics in the validation cohort were well balanced in comparison with the derivation cohort (all *P* > .05). During the median follow‐up of 42.0 months, 112 patients (53.8%) experienced HCC recurrence. DFS at 3 and 5 years were 41.7% and 31.5%, respectively. There was no significant difference in DFS between the derivation and validation cohorts (*P* = .711 by log‐rank test).

**Figure 4:**
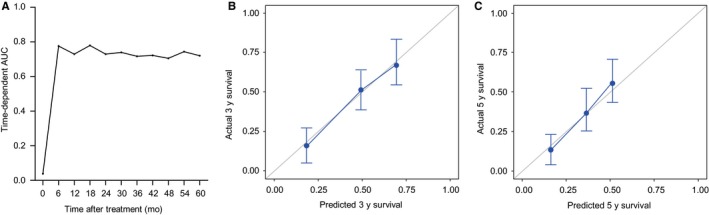
Performance power of nomogram in the validation cohort. (A) AUCs for the prediction of DFS at 6‐60 months after RFA among the validation cohort. (B) Calibrations of the nomogram for the prediction of DFS at 3 years among the validation cohort. (C) Calibrations of the nomogram for the prediction of DFS at 5 years among the validation cohort

The discriminative ability of the nomogram among the validation cohort was similar in terms of iAUC metrics to predict DFS at 6‐60 months after RFA (Figure 4A), and agreement of the predicted and actual DFS at 3 years (Figure 4B) and 5 years (Figure 4C). The overall c‐index of the prognostic nomogram was 0.748 (95% CI 0.694‐0.803), which was also higher than those of the conventional staging or scoring systems (Table S3). The median DFS of low‐, intermediate‐, and high‐risk groups were 112.2, 34.8, and 11.6 months, respectively (*P* < .001 by log‐rank test, Figure 3B).

### 3.4 Prognostic nomogram incorporating NLR and ALBI grade

We also investigated whether NLR and ALBI grade rather than simple lymphocyte count or albumin level have discriminative ability in predicting DFS. There was no statistical differences in NLR and ALBI grade between the validation cohort and derivation cohort (Table S4). On univariate analysis, NLR and ALBI grade were associated with DFS in the derivation cohort, which was entered into a multivariate Cox‐regression model (Table S5). On multivariate analysis, tumor size, tumor number, AFP level, PIVKA‐II level, presence of ascites, NLR, and ALBI grade were identified as prognostic factors with statistical significance (Table S6). The prognostic nomogram was generated based on the total sum of the risk scores (Figure S1). The nomogram showed a good discrimination capability to predict DFS at 6‐60 months after RFA, with c‐index of 0.755 for the derivation cohort and 0.746 for the validation cohort, respectively. When patients were stratified into three groups based on the sum of the risk scores, DFS was clearly separated both on the derivation and validation cohorts (Figure S2).

## 4 DISCUSSION

Nomograms are widely used as prognostic tools in routine clinical practice since they have the ability to fulfill desire toward personalized medicine through integrating diverse prognostic and determinant variables.^20^ In the current study, based on the previous experience that large variations exist in clinical outcomes among patients with HCC undergoing RFA as first‐line treatment,^21^ we aimed to develop and validate a novel prognostic nomogram optimized for such patients through large patient cohorts. As a matter of fact, current staging or scoring systems have hardly discriminated the individual patient's prognosis because they did not fully appreciate the heterogeneity of HCC patients treated with RFA.^22^ By a novel nomogram using the tumor characteristics, hepatic functional reserve, and host's immunological factor, accurate stratification of HCC patients treated with RFA into distinct prognostic subgroups might be available, providing a better discriminatory ability compared to other staging or scoring systems.

Our study has several strengths. First, even though there have been several studies concerning the prognostic model for HCC patients treated with RFA to overcome the disadvantages of conventional staging or scoring systems,^23^, ^24^, ^25^ they were primarily limited by inadequate external validation, which is a critical component to confirm the prognostic system. So, we tried to recruit an independent cohort during the same study period to support the clinical relevance of our proposed nomogram. In addition, the large sample size of more than 700 patients in the derivation cohort may have further improved the statistical power, compared to the previous study.^22^ Second, the easy‐to‐use graphical tool consists of variables routinely evaluated in clinics such as tumor size, tumor number, AFP level, PIVKA‐II level, lymphocyte count, albumin level, and presence of ascites. Therefore, additional costly tests may not be necessary. Notably, we suggest that PIVKA‐II level is independently associated with prognosis. To date, the prognostic role of PIVKA‐II, especially for patients treated with RFA, has remained elusive.^4^, ^19^, ^26^, ^27^ However, our study provides clear evidence on the value of measuring PIVKA‐II level at baseline for risk stratification. Given that its weight may be larger than that of AFP, but less than that of tumor size or number, PIVKA‐II level might be used as an ancillary method to complement conventional tumor factors for delicate prognostification. Furthermore, it is noteworthy that lymphopenia has been identified as a prognostic factor in HCC. To date, in other solid tumors including breast, renal, colorectal, prostate, and bladder cancer, lymphopenia has been proposed as a poor prognostic factor.^28^ As lymphocytes are a crucial part of antitumor immunity, lymphopenia can be a surrogate marker of tumor‐induced immune suppression. To our knowledge, this is the first study to address the role of lymphopenia in HCC patients treated with RFA. In a similar context, the benefit of adjuvant autologous T‐lymphocyte‐based immunotherapy to improve post‐RFA prognosis had already been proven through a phase III trial,^29^ and more recently, a phase III clinical trial to assess the beneficial effect of adjuvant immune checkpoint inhibitor which can enhance the host's T‐cell activity (NCT03383458) is still ongoing. Another hypothesis explaining the prognostic role of lymphopenia might be suggested. In the setting of underlying liver cirrhosis and portal hypertension, increased neutrophil and monocyte counts and decreased lymphocyte count might reflect the severity of liver disease,^30^, ^31^ which is closely associated with de novo HCC in the background liver.^32^ Intriguingly, NLR was also found to have prognostic power similar to that observed in other studies.^33^, ^34^, ^35^ Other variables of our nomogram, which are tumor size, tumor number, AFP, hypoalbuminemia, and ascites, also have sufficient scientific rationale in determining patients' prognosis, consistent with the previous literature.^36^, ^37^, ^38^ We also validated that ALBI grade can independently discriminate patients' prognosis in the setting of RFA.

When patients were stratified into low‐, intermediate‐, and high‐risk groups, the median DFS rates were 106.3, 33.6, and 9.3 months, respectively. Hence, our proposed nomogram can be utilized in routine clinical practice in establishing optimal surveillance protocols, guiding treatment strategies, identifying potential candidates for effective adjuvant therapy, or designing future clinical trials. For example, local ablation might be preferred to hepatic resection in terms of its less invasiveness for the low‐risk group, whereas hepatic resection might be preferred in terms of better tumor control for intermediate‐ or high‐risk groups. Furthermore, in order to maximize the therapeutic benefit of costly adjuvant immunotherapy and to optimize the number of patients needed to be treated, high‐risk group might be considered reasonable candidates for such adjuvant immunotherapy.

Although our study attempted to overcome the shortcomings of previous studies, several unresolved limitations exist. First, this is an observational study which includes single‐ethnic population in a HBV‐endemic geographic area, which is somewhat subject to selection bias. However, to confirm the reliability of our study with robust evidence, we tried to conduct external validation in an independent cohort where the variables were well balanced in comparison to those of the derivation cohort. Furthermore, since there has been no evidence of a relationship between the ethnicity or the etiology and the prognosis of HCC patients treated with RFA,^25^ this nomogram may be widely applicable. Furthermore, this cohort did not include patients treated with the newer ablation methods such as microwave ablation or irreversible electrophoration.^8^, ^39^, ^40^ Since such methods to treat early‐stage HCC have not yet been popular in the Republic of Korea, further studies are required to keep pace with the latest up‐to‐date knowledge.

In conclusion, a simple individualized nomogram optimized for treatment‐naïve HCC patients undergoing RFA as a first‐line treatment allowed more delicate prognostification in terms of HCC recurrence, with the appropriate verification in an independent cohort. This novel prognostic calculator can serve as a useful instrument for not only guiding surveillance schedules and treatment strategies but also designing future prospective clinical trials.

## 5 ETHICS STATEMENT

The study was performed in accordance with the Declaration of Helsinki and Good Clinical Practice and was approved by the institutional review boards (4‐2018‐0969). Informed consent for the invasive procedures was acquired from all patients.

## CONFLICT OF INTEREST

None declared.

## AUTHOR CONTRIBUTIONS

All authors were involved in drafting the article or revising it critically for important intellectual content, and all authors approved the final version to be submitted for publication. Chang Gon Kim and Beom Kyung Kim designed the study. Hyun Woong Lee, Hye Jin Choi, Jung Il Lee, Hye Won Lee, Seung Up Kim, Jun Yong Park, Do Young Kim, Kwang‐Hyub Han, Han Sang Kim, Kwan Sik Lee, Gyoung Min Kim, Man Deuk Kim, Jong Yoon Won, Do Yun Lee, and Beom Kyung Kim were involved in data acquisition. Chang Gon Kim, Kyung Hwan Kim, Seong Jin Choi, Yongun Kim, and Beom Kyung Kim were involved in data analysis. Chang Gon Kim and Beom Kyung Kim wrote the manuscript. Beom Kyung Kim reviewed, revised, and gave final approval of the manuscript. The results presented in this paper have not been published previously in their entirety or in part, except in abstract form.
