# Supplementary figures and images for: Development and validation of a prognostic model for patients with hepatocellular carcinoma undergoing radiofrequency ablation
Source: Cancer Med. 2019 Jul 10;8(11):5023–32. doi: 10.1002/cam4.2417 (PMC6718586; doi:10.1002/cam4.2417)

Supplementary Figure S1

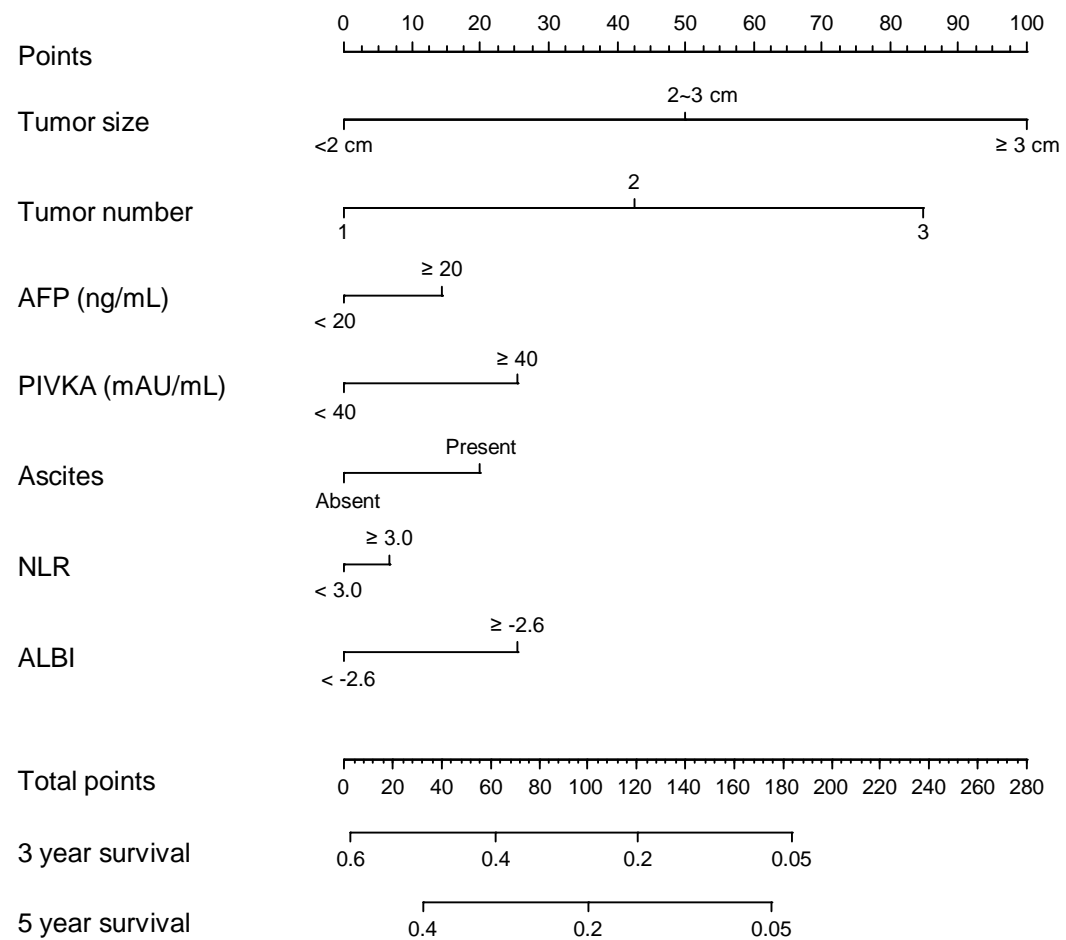

Supplementary Figure S2

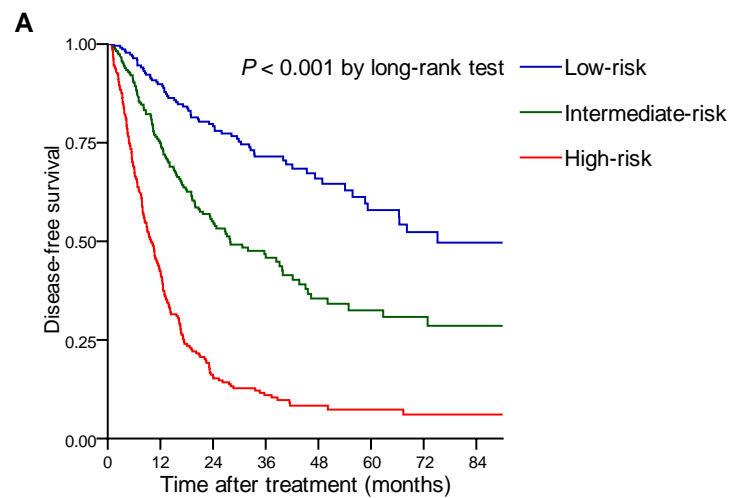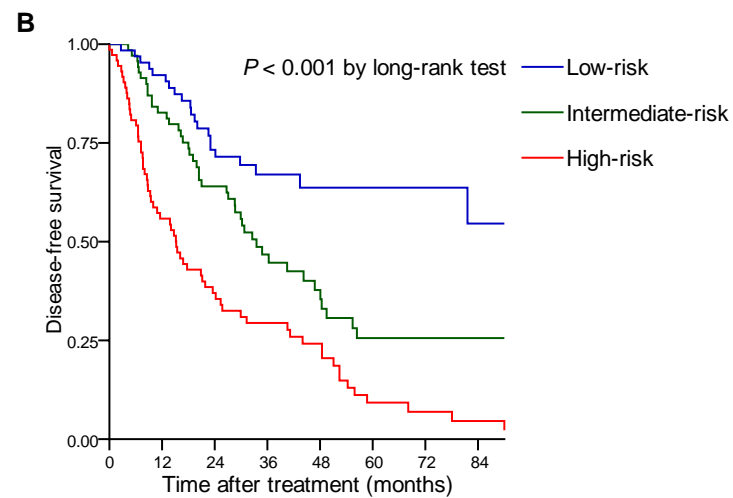

Supplement: Supplementary file 1 [file CAM4-8-5023-s001.pdf]
