# Supplementary material for: Development and validation of a prognostic model for patients with hepatocellular carcinoma undergoing radiofrequency ablation
Source: Cancer Med. 2019 Jul 10;8(11):5023–32. doi: 10.1002/cam4.2417 (PMC6718586; doi:10.1002/cam4.2417)
Supplement: Supplementary file 2 [file CAM4-8-5023-s002.docx]

| Staging or scoring systems | | Derivation cohort (n = 757) | Validation cohort (n = 208) | *P* value |
| --- | --- | --- | --- | --- |
| AJCC eighth edition | |  |  | 0.738 |
|  | I | 644 (85.1%) | 175 (84.1%) |  |
|  | II | 113 (14.9%) | 33 (15.9%) |  |
| BCLC | |  |  | 0.500 |
|  | 0 | 375 (49.5%) | 98 (47.1%) |  |
|  | A | 292 (38.6%) | 89 (42.8%) |  |
|  | B | 90 (11.9%) | 21 (10.1%) |  |
| CLIP | |  |  | 0.589 |
|  | 0 | 437 (57.7%) | 115 (55.3%) |  |
|  | 1 | 251 (33.2%) | 71 (34.1%) |  |
|  | 2 | 60 (7.9%) | 21 (10.1%) |  |
|  | 3 | 9 (1.2%) | 1 (0.5%) |  |
| CUPI | |  |  | 0.770 |
|  | Low risk | 752 (99.3%) | 207 (99.5%) |  |
|  | Intermediate risk | 5 (0.7%) | 1 (0.5%) |  |
| Okuda | |  |  | 0.780 |
|  | I | 617 (81.5%) | 166 (79.8%) |  |
|  | II | 138 (18.2%) | 41 (19.7%) |  |
|  | III | 2 (0.3%) | 1 (0.5%) |  |
| JIS | |  |  | 0.820 |
|  | 0 | 575 (76.0%) | 154 (74.0%) |  |
|  | 1 | 164 (21.7%) | 48 (23.1%) |  |
|  | 2 | 18 (2.4%) | 6 (2.9%) |  |
| GETCH | |  |  | 0.953 |
|  | Low risk | 548 (72.4%) | 151 (72.6%) |  |
|  | Intermediate risk | 209 (27.6%) | 57 (27.4%) |  |

**Supplementary Table S1.** Distribution of patients according to conventional staging or scoring systems

Abbreviations: AJCC, American Joint Committee on Cancer; BCLC, Barcelona Clinic Liver Center; CLIP, Cancer of the Liver Italian Program; CUPI, Chinese University Prognostic Index; JIS, Japan Integrated Staging; GETCH ,Group d’Etude ed te Traitement du Carcinome Hepatocellular.

**Supplementary Table S2.** C-index of seven conventional staging or scoring systems among the derivation cohort

| Staging or scoring systems | C-index | 95% CI |
| --- | --- | --- |
| AJCC eighth edition | 0.569 | 0.552-0.586 |
| BCLC | 0.683 | 0.656-0.710 |
| CLIP | 0.626 | 0.600-0.652 |
| CUPI | 0.505 | 0.501-0.508 |
| Okuda | 0.559 | 0.539-0.579 |
| JIS | 0.605 | 0.584-0.626 |
| GETCH | 0.555 | 0.532-0.579 |

Abbreviations: C-index, concordance-index; CI, confidence interval; AJCC, American Joint Committee on Cancer; BCLC, Barcelona Clinic Liver Center; CLIP, Cancer of the Liver Italian Program; CUPI, Chinese University Prognostic Index; JIS, Japan Integrated Staging; GETCH, Group d’Etude ed te Traitement du Carcinome Hepatocellular.

| Staging or scoring systems | C-index | 95% CI |
| --- | --- | --- |
| AJCC eighth edition | 0.581 | 0.549-0.612 |
| BCLC | 0.635 | 0.587-0.684 |
| CLIP | 0.617 | 0.570-0.664 |
| CUPI | 0.504 | 0.498-0.509 |
| Okuda | 0.556 | 0.520-0.592 |
| JIS | 0.590 | 0.551-0.629 |
| GETCH | 0.552 | 0.512-0.593 |

**Supplementary Table S3.** C-index of seven conventional staging or scoring systems among the validation cohort

Abbreviations: C-index, concordance-index; CI, confidence interval; AJCC, American Joint Committee on Cancer; BCLC, Barcelona Clinic Liver Center; CLIP, Cancer of the Liver Italian Program; CUPI, Chinese University Prognostic Index; JIS, Japan Integrated Staging; GETCH, Group d’Etude ed te Traitement du Carcinome Hepatocellular.

**Supplementary Table S4.** Neutrophil-to-lymphocyte ratio and albumin-bilirubin grade of patients from derivation and validation cohorts

| Variables | | Derivation cohort (n = 757) | Validation cohort (n = 208) | *P* value |
| --- | --- | --- | --- | --- |
| NLR | |  |  | 0.770 |
|  | < 3.0 | 586 (77.4%) | 163 (78.4%) |  |
|  | ≥ 3.0 | 171 (22.6%) | 45 (21.6%) |  |
| ALBI grade | |  |  | 0.458 |
|  | < -2.6 | 375 (49.5%) | 97 (46.6%) |  |
|  | ≥ -2.6 | 382 (50.5%) | 111 (53.4%) |  |

Abbreviations: NLR, neutrophil-to-lymphocyte ratio; ALBI, albumin-bilirubin.

**Supplementary Table S5.** Factors associated with disease-free survival in the derivation cohort including neutrophil-to-lymphocyte ratio and albumin-bilirubin grade

| Variables | | | | Univariate analysis | |  | Multivariate analysis | |
| --- | --- | --- | --- | --- | --- | --- | --- | --- |
|  |  |  |  | HR (95% CI) | *P* value |  | Adjusted HR (95% CI) | *P* value |
| Age | | |  | | 0.229 |  |  |  |
|  | < 65 years | | Ref | |  |  |  |  |
|  | ≥ 65 years | | 1.129 (0.927-1.375) | |  |  |  |  |
| Sex | | |  | | 0.239 |  |  |  |
|  | Male | | 1.151 (0.911-1.454) | |  |  |  |  |
|  | Female | | Ref | |  |  |  |  |
| Etiology | | |  | | 0.008 |  |  | 0.139 |
|  | HCV | | 1.338 (1.090-1.768) | |  |  | 1.214 (0.939-1.570) |  |
|  | Non-HCV | | Ref | |  |  | Ref |  |
| Tumor size | | |  | | <0.001 |  |  | <0.001 |
|  | | < 2 cm | Ref | |  |  | Ref |  |
|  | 2 ~ 3 cm | | 2.061 (1.654-2.569) | |  |  | 1.861 (1.484-2.333) |  |
|  | ≥ 3 cm | | 6.562 (4.958-8.686) | |  |  | 5.265 (3.921-7.070) |  |
| Tumor numbers | | |  | | <0.001 |  |  | <0.001 |
|  | | 1 | Ref | |  |  | Ref |  |
|  | 2 | | 2.291 (1.705-3.077) | |  |  | 1.531 (1.121-2.091) |  |
|  | 3 | | 4.887 (3.337-7.158) | |  |  | 3.969 (2.669-5.903) |  |
| AFP | | |  | | <0.001 |  |  | <0.001 |
|  | | < 20 ng/mL | Ref | |  |  | Ref |  |
|  | ≥ 20 ng/mL | | 1.595 (1.307-1.946) | |  |  | 1.496 (1.214-1.844) |  |
| PIVKA-II | | |  | | <0.001 |  |  | <0.001 |
|  | | < 40 mAU/mL | Ref | |  |  | Ref |  |
|  | ≥ 40 mAU/mL | | 1.980 (1.625-2.412) | |  |  | 1.494 (1.212-1.841) |  |
| Anemia | | |  | | <0.001 |  |  | 0.235 |
|  | | Presence | 1.830 (1.493-2.242) | |  |  | 1.150 (0.913-1.449) |  |
|  | Absence | | Ref | |  |  | Ref |  |
| Platelet | | |  | | 0.009 |  |  | 0.703 |
|  | | < 150 x 10^3^ /uL | 1.366 (1.080-1.728) | |  |  | 1.051 (0.815-1.356) |  |
|  | ≥ 150 x 10^3^ /uL | | Ref | |  |  | Ref |  |
| Ascites | | |  | | <0.001 |  |  | 0.001 |
|  | | Presence | 1.740 (1.317-2.300) | |  |  | 1.651 (1.228-2.221) |  |
|  | Absence | | Ref | |  |  | Ref |  |
| AST | | |  | | <0.001 |  |  | 0.378 |
|  | | < 40 IU/L | Ref | |  |  | Ref |  |
|  | ≥ 40 IU/L | | 1.742 (1.425-2.130) | |  |  | 1.115 (0.875-1.420) |  |
| ALT | | |  | | 0.013 |  |  | 0.631 |
|  | | < 40 IU/L | Ref | |  |  | Ref |  |
|  | | ≥ 40 IU/L | 1.359 (1.066-1.733) | |  |  | 1.071 (0.810-1.414) |  |
| ALP | | |  | | <0.001 |  |  | 0.349 |
|  | | < 143 IU/L | Ref | |  |  | Ref |  |
|  | ≥ 143 IU/L | | 2.225 (1.626-3.046) | |  |  | 1.179 (0.835-1.665) |  |
| NLR | | |  | | <0.001 |  |  | 0.014 |
|  | | < 3.0 | Ref | |  |  | Ref |  |
|  | ≥ 3.0 | | 1.592 (1.281-1.979) | |  |  | 1.339 (1.062-1.689) |  |
| ALBI grade | | |  | | <0.001 |  |  | 0.041 |
|  | | < -2.6 | Ref | |  |  | Ref |  |
|  | ≥ -2.6 | | 2.167 (1.766-2.659) | |  |  | 1.303 (1.011-1.679) |  |

Abbreviations: HR, hazard ratio; CI, confidence interval; Ref, reference; HCV, hepatitis C virus; AFP, alpha-fetoprotein; PIVKA-II, prothrombin induced by vitamin K absence-II; AST, aspartate aminotransferase; ALT, alanine aminotransferase; ALP, alkaline phosphatase; NLR, neutrophil-to-lymphocyte ratio; ALBI, albumin-bilirubin.

| Variables | | | ß-coefficient | *P* value | Risk score |
| --- | --- | --- | --- | --- | --- |
| Tumor size | | |  | <0.001 |  |
|  | | < 2 cm | Ref |  | 0 |
|  | 2 ~ 3 cm | | 0.627 |  | 50 |
|  | ≥ 3 cm | | 1.679 |  | 100 |
| Tumor numbers | | |  | <0.001 |  |
|  | | 1 | Ref |  | 0 |
|  | 2 | | 0.478 |  | 42 |
|  | 3 | | 1.406 |  | 84 |
| AFP | | |  | <0.001 |  |
|  | | < 20 ng/mL | Ref |  | 0 |
|  | ≥ 20 ng/mL | | 0.447 |  | 15 |
| PIVKA-II | | |  | <0.001 |  |
|  | | < 40 mAU/mL | Ref |  | 0 |
|  | ≥ 40 mAU/mL | | 0.428 |  | 26 |
| Ascites | | |  | 0.001 |  |
|  | | Presence | 0.496 |  | 0 |
|  | Absence | | Ref |  | 20 |
| NLR | | |  | 0.007 |  |
|  | | < 3.0 | Ref |  | 0 |
|  | ≥ 3.0 | | 0.309 |  | 8 |
| ALBI | | |  | <0.001 |  |
|  | < -2.6 | | Ref |  | 0 |
|  | ≥ -2.6 | | 0.405 |  | 25 |

**Supplementary Table S6.** ß-coefficient and risk score from multivariate Cox-regression model in the derivation cohort including neutrophil-to-lymphocyte ratio and albumin-bilirubin grade

Abbreviations: Ref, reference; AFP, alpha-fetoprotein; PIVKA-II, prothrombin induced by vitamin K absence-II; NLR, neutrophil-to-lymphocyte ratio; ALBI, albumin-bilirubin.
